# The Enhanced Pneumococcal LAMP Assay: A Clinical Tool for the Diagnosis of Meningitis Due to *Streptococcus pneumoniae*

**DOI:** 10.1371/journal.pone.0042954

**Published:** 2012-08-10

**Authors:** Dong Wook Kim, Paul E. Kilgore, Eun Jin Kim, Soon Ae Kim, Dang Duc Anh, Bai Qing Dong, Jung Soo Kim, Mitsuko Seki

**Affiliations:** 1 Department of Pharmacy, College of Pharmacy, Hanyang University, Ansan, Republic of Korea; 2 Molecular Bacteriology Laboratory, International Vaccine Institute, Seoul, Republic of Korea; 3 Translational Research Division, International Vaccine Institute, Seoul, Republic of Korea; 4 Wayne State University, Eugene Applebaum College of Pharmacy and Health Sciences, Department of Pharmacy Practice, Detroit, Michigan, United States of America; 5 National Institute of Hygiene and Epidemiology, Hanoi, Vietnam; 6 Guangxi Zhuang Autonomous Region, Center for Disease Prevention and Control, Nanning, Guangxi, People’s Republic of China; 7 Chonbuk National University School of Medicine, Jeonju, Republic of Korea; 8 Department of Oral Health Sciences, Nihon University School of Dentistry, Tokyo, Japan; 9 Dental Research Center, Nihon University School of Dentistry, Tokyo, Japan; Health Protection Agency, United Kingdom

## Abstract

**Background:**

*Streptococcus pneumoniae* is a leading cause of invasive bacterial disease in developed and developing countries. We studied the loop-mediated isothermal amplification (LAMP) technique to assess its suitability for detecting *S. pneumoniae* nucleic acid in cerebrospinal fluid (CSF).

**Methodology/Principal Findings:**

We established an improved LAMP assay targeting the *lytA* gene (*Streptococcus pneumoniae* [Sp] LAMP). The analytical specificity of the primers was validated by using 32 reference strains (10 *Streptococcus* and seven non-*Streptococcus* species) plus 25 clinical alpha-hemolytic streptococcal strains, including four *S. pneumoniae* strains and 21 other strains (3 *S. oralis*, 17 *S. mitis*, and one *Streptococcus* species) harboring virulence factor-encoding genes (*lytA* or *ply*). Within 30 minutes, the assay could detect as few as 10 copies of both purified DNA and spiked CSF specimens with greater sensitivity than conventional polymerase chain reaction (PCR). The linear determination range for this assay is 10 to 1,000,000 microorganisms per reaction mixture using real-time turbidimetry. We evaluated the clinical sensitivity and specificity of the Sp LAMP assay using 106 randomly selected CSF specimens from children with suspected meningitis in Korea, China and Vietnam. For comparison, CSF specimens were also tested against conventional PCR and culture tests. The detection rate of the LAMP method was substantially higher than the rates of PCR and culture tests. In this small sample, relative to the LAMP assay, the clinical sensitivity of PCR and culture tests was 54.5% and 33.3%, respectively, while clinical specificity of the two tests was 100%.

**Conclusions/Significance:**

Compared to PCR, Sp LAMP detected *S. pneumoniae* with higher analytical and clinical sensitivity. This specific and sensitive LAMP method offers significant advantages for screening patients on a population basis and for diagnosis in clinical settings.

## Introduction

*Streptococcus pneumoniae* is associated with invasive and noninvasive diseases, such as meningitis, bacteremia, septicemia, community-acquired pneumonia, and otitis media [1]. Each year, approximately 875,000 children die as a result of pneumococcal disease, with most deaths occurring in developing countries [2]. Elderly persons and immunocompromised hosts (including patients with HIV infection, sickle-cell anemia and other chronic diseases) also bear a substantial burden of pneumococcal disease in developed and developing countries [1]. Although traditional antimicrobial therapy has proven to be effective, pneumococcal resistance to essential antimicrobials has increased rapidly in recent decades and poses a serious global problem [3].

For several decades, diagnosis of pneumococcal infection has relied on classic bacterial culture and identification methods. However, isolation and identification of pneumococcus are complicated by contamination with alpha-hemolytic streptococci (*S. mitis* and *S. oralis*) belonging to normal human flora. *S. mitis* and *S. oralis* are most closely related to pneumococcus on this ecological basis, and their 16S rRNA sequences share over 99% identity with pneumococcus, which presents a challenge for clinical laboratories to differentiate pneumococcus from other alpha-hemolytic oral streptococci [4]. Such misidentification might affect diagnosis and treatment. In developing countries, accurate diagnosis of pneumococcus remains a challenge due to the limited availability of routine microbiology laboratory services and injudicious use of antimicrobial agents.

Molecular assays are inherently valuable because of their enhanced analytical and clinical sensitivity and specificity. Such assays also have the potential to provide a diagnosis by detecting nonviable organisms in patients who have received antibiotic treatment [5], [6], [7]. The development of PCR assays that target *ply* [5] and *lytA* (Sp PCR) [6] genes was an important milestone in the evolution of laboratory diagnosis of pneumococcus. However, the *ply* and *lytA* genes, initially thought to be present only in *S. pneumoniae*, were recently reported in strains of *S. mitis* [4]. Thus, PCR-based detection that targets those genes might result in over-detection of pneumococcus [8]. A real-time PCR method that targets the *lytA* gene was reported to be sensitive and specific for detecting *S. pneumoniae* [9]. However, PCR and real-time PCR equipment is relatively expensive, and the assays are complex to perform in resource-limited laboratories in developing countries.

Detection of *S. pneumoniae* teichoic acid antigen in urine (Binax NOW® *S. pneumoniae*, Alere International Limited, Galway, Ireland) is an alternative to culture or PCR; however, its specificity is low in children because it can give positive results in healthy children who carry pneumococci and other closely related *Streptococcus* species [10].

In 2000, a novel nucleic acid detection method, loop-mediated isothermal amplification (LAMP), was first reported by Notomi and colleagues [11]. LAMP employs a DNA polymerase with strand-displacement activity, along with two inner primers (forward inner primer, FIP; backward inner primer, BIP) and two outer primers (F3, B3) that recognize six separate regions within a target DNA sequence. This method utilizes a unique priming mechanism that yields specific DNA products in a shorter time than PCR. Additional primers (i.e., the so-called loop primers: loop-primer forward, LF; loop-primer backward, LB) designed to anneal the loop structure in LAMP can be used to accelerate the LAMP reaction resulting in enhanced sensitivity [12]. The loop primers are now commonly used in practical applications of LAMP [13]. Although LAMP assays for detecting pneumococcus in oral bacteria have been established [14], their efficacy in diagnosing bacterial meningitis has not yet been tested [15].

In this study, we established an improved LAMP assay by adding a loop primer targeting the *lytA* gene (Sp LAMP), and we compared the detection performance of the Sp LAMP assay with that of Sp PCR by using cerebrospinal fluid (CSF) specimens from patients with suspected meningitis in Korea, China and Vietnam [16]. To our knowledge, this is the first report of using a LAMP assay for detecting *S. pneumoniae* in clinical CSF specimens.

**Figure 1:**
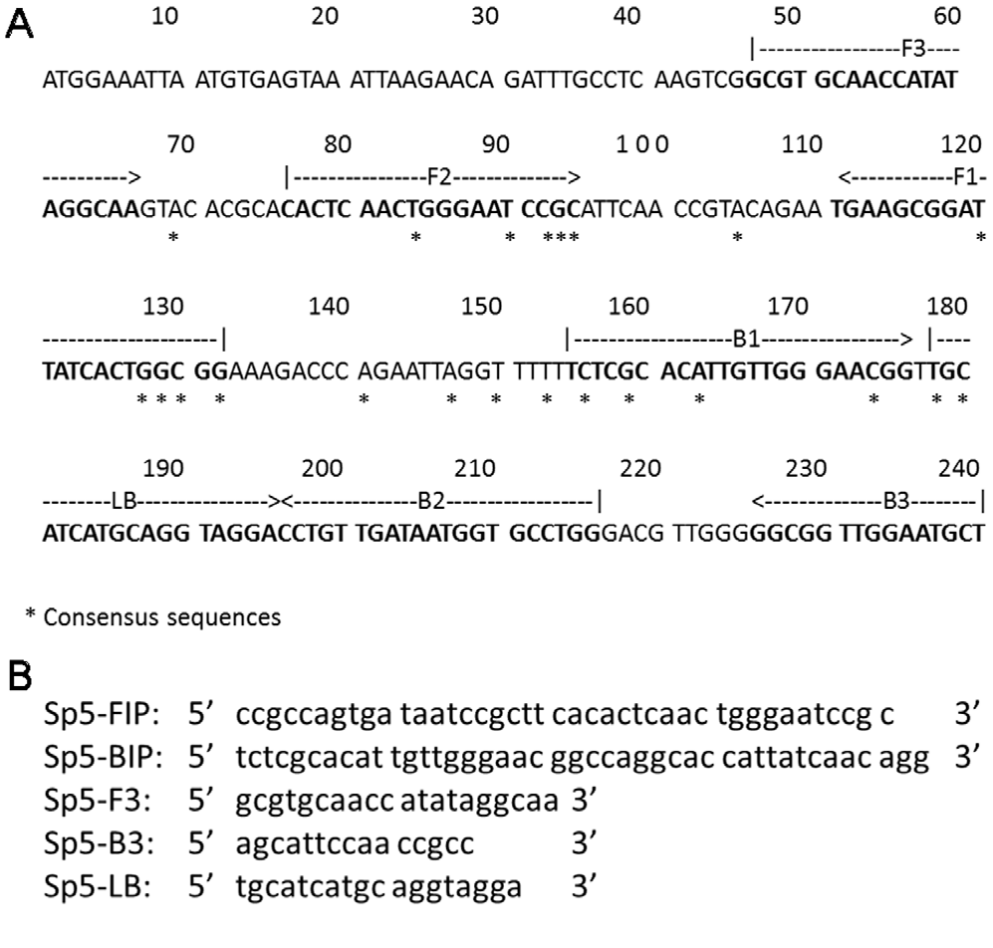
Nucleotide sequence of the *lytA* gene used to design the Sp LAMP primer. The sequences used for Sp LAMP primers are indicated by arrows (A). Structure and sequence of the primers used in the Sp LAMP reaction (B).

## Methods

### Ethics Statement

To analyze the specimens in this study, we utilized CSF specimens preserved from our previous surveillance study [16]. All CSF specimens utilized in this study were de-identified prior to laboratory processing and analysis. Ethical approvals for patient specimen collection during surveillance were obtained from the following institutions: International Vaccine Institute, Seoul, Korea; Harbor UCLA Medical Center, Torrance, CA, USA; Chonbuk National University Hospital, Jeonju, Korea; Chonju Presbyterian Hospital, Jeonju, Korea; Namwon Medical Center, Namwon, Korea; Jeongeub Asan Foundation Hospital, Jeongeub, Korea; Won Kwan University Hospital, Iksan, Korea; National Institute of Hygiene and Epidemiology, Hanoi, Vietnam; National Institute of Pediatrics, Hanoi, Vietnam; St. Paul Hospital, Hanoi, Vietnam; Bach Mai Hospital, Hanoi, Vietnam; and the Guangxi Zhuang Autonomous Region Center for Disease Control, Nanning, China. Each institution participated in prospective, population-based surveillance for childhood meningitis from 1999 to 2002. During those surveillance studies, written consent was not obtained as the collection of CSF was considered routine standard care for hospitalized children with suspected bacterial meningitis. For this reason, verbal consent of the parent or legal guardian present with the child during the hospitalization was recorded in the patient’s medical chart at the time of the clinical lumbar puncture procedure. This consenting procedure was approved by the local scientific ethics review committees of participating institutions.

**Figure 2:**
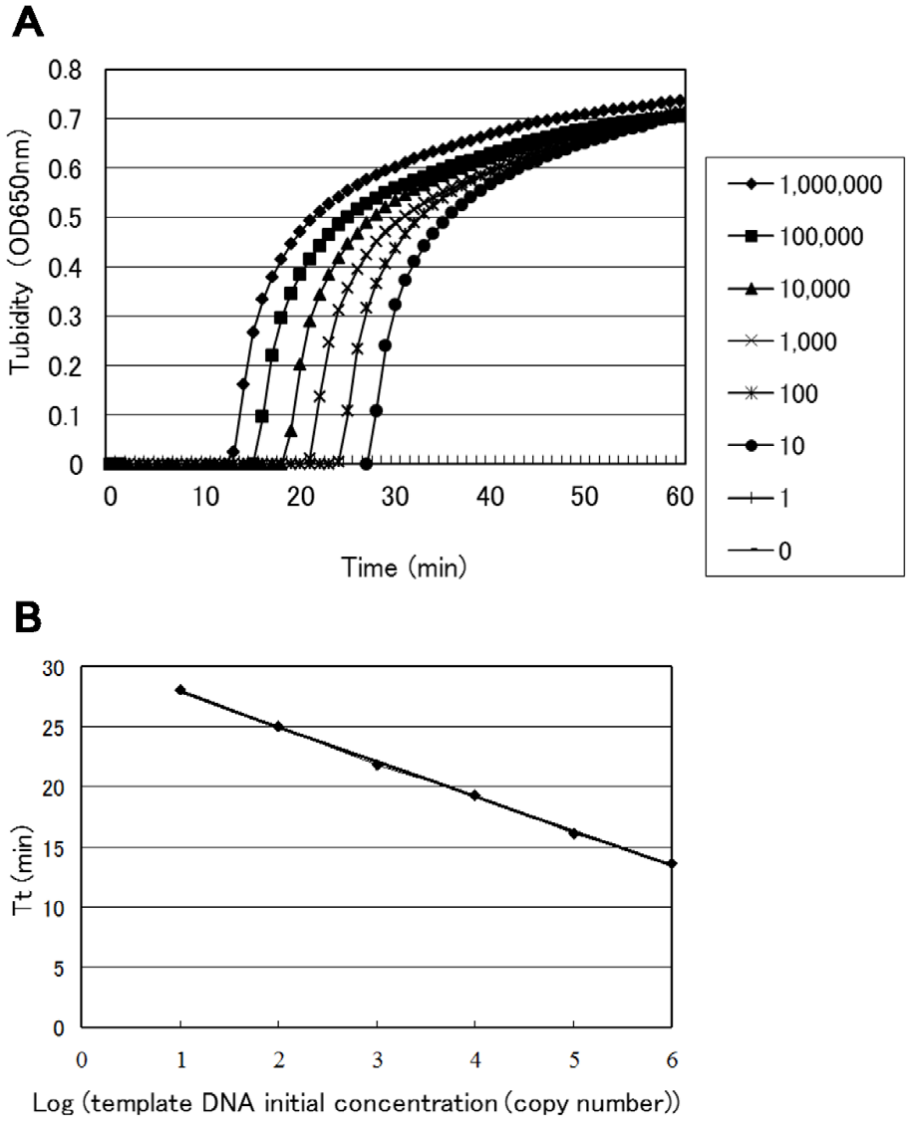
Real-time sensitivity of Sp LAMP, as monitored by the measurement of turbidity. Shown from left to right in the figure are the curves of decreasing concentration (1,000,000 to 1) of bacteria. The detection limit was 10 copies (A). The relationship between the threshold time (*Tt*) of each sample and the log of the amount of initial template DNA (B).

**Figure 3:**
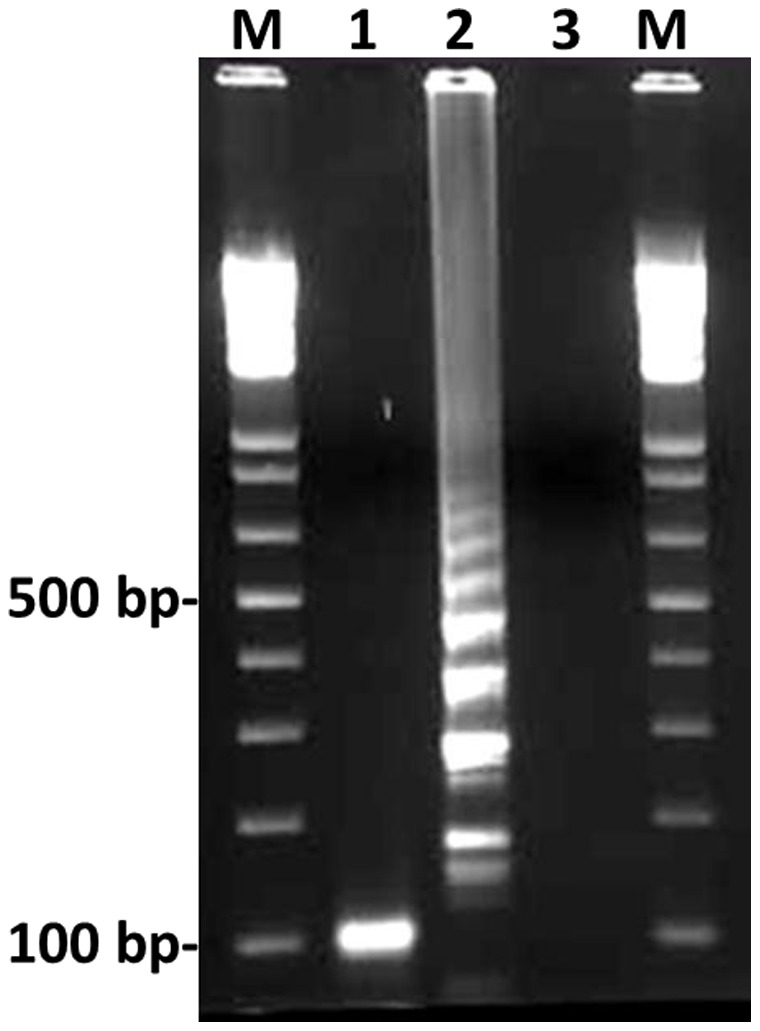
Electrophoretic analysis of Sp LAMP-amplified products. Lane M, 100-bp ladder (New England Biolabs, Beverly, MA, USA) used as a size marker; lane 1, Sp LAMP product from lane 2 after digestion with TasI (Fermentas, Inc.). The digested fragments were 107 and 108 bp; lane 2, 10^6^ copies of the genomic DNA of *S. pneumoniae* ATCC 6305; lane 3, no template.

### Clinical CSF Specimens

Children with suspected meningitis who were less than five years old were prospectively enrolled at study hospitals [16]. Clinicians classified patients as having suspected, probable or confirmed bacterial meningitis based on clinical signs and symptoms, CSF parameters (e.g., white blood cell count, glucose and protein levels) and bacterial testing (i.e., culture and latex agglutination testing). CSF was streaked on commercial blood agar culture medium (Becton, Dickinson & Co., Franklin Lakes, NJ, USA) and incubated in 5% CO_2_ at 37°C for 3 days and checked daily for growth in the previous study [16]. Isolates were identified using standard microbiological criteria [17]. To compare pneumococcal detection by PCR and LAMP, 106 CSF specimens (18 Sp PCR-positive and 88 Sp PCR-negative) were randomly selected from CSF collected between 1998 and 2002 in the prospective study [16] of bacterial meningitis in Korea (n = 40), China (n = 33) and Vietnam (n = 33). CSF specimens were pretreated at 95°C for 2 min and centrifuged (13,000×*g*, 5 min). The supernatant was saved for Sp PCR and Sp LAMP analysis. Sp PCR was performed as described previously [7]. A 5-µl aliquot (a sample volume consistent with future clinical applications) from each CSF specimen was subjected to Sp PCR (25-µl reaction volume) and Sp LAMP, as described below.

**Table 1:** Detection of *S. pneumoniae* in 25 clinical isolates of oral streptococci by PCR and LAMP methods.

Number of isolates	Results of API test^a^	Optochin^b^	Bile test^c^	Identification^d^	PCR^e^		LAMP^e^
					*ply*	*lytA*	
1, 2	*S. oralis*	−	+	*S. oralis*	+	−	−
3	*S. oralis*	−	−	*S. oralis*	+	−	−
4 to 14	*S. mitis*	−	+	*S. mitis*	+	−	−
15 to 17	*S. mitis*	−	−	*S. mitis*	+	−	−
18	*S. mitis*	−	+	*S. mitis*	−	+	−
19, 20	*S. mitis*	−	−	*S. mitis*	−	+	−
21	Not identified	−	−	*Streptococcus* species	−	+	−
22 to 25	*S. pneumonia*	+	+	*S. pneumoniae*	+	+	+

a Classification based on API20 Strep identification testing.

b +, optochin sensitive; −, optochin resistant.

c +, bile soluble; −, bile insoluble.

d Final identification from API20 strep testing, optochin sensitivity and bile solubility.

e +, amplification occurred; −, amplification did not occur.

### Bacterial Strains

Overall, 32 standard reference strains representing 10 *Streptococcus* species and seven non-*Streptococcus* species were evaluated. Among the seven non-*Streptococcus* species were *Haemophilus influenzae* (RD), *Escherichia coli* (DH5α), *Actinobacillus actinomycetemcomitans* (Y-4), *Porphyromonas gingivalis* (381, ATCC49417, ATCC33277), *Actinomyces naeslundii* (WVU627, T14, ATCC12104), *Prevotella intermedia* (ATCC25611), and *P. nigrescens* (ATCC25261). The 10 *Streptococcus* species were *Streptococcus mitis* (ATCC903), *S. oralis* (ATCC9811, 10557), *S. gordonii* (ATCC12396), *S. agalactiae* (IID1625), *S. milleri* (NCTC10703), *S. sobrinus* (NIDR6715, OMZ176), *S. mutans* (XC47, PK1, JC2), *S. sanguis* (ATCC10556), *S. salivarius* (ATCC7073, ATCC9222, HHT), and *S. pneumoniae* (R6, ATCC6305, GTC261, IID553, IID554). In addition, 25 clinical alpha-hemolytic streptococcal strains were evaluated, including four *S. pneumoniae* strains and 21 other *Streptococcus* species (three *S. oralis*, 17 *S. mitis*, and one *Streptococcus* species) known to carry the *ply* and *lytA* genes [14].

**Table 2:** Detection limits of the LAMP and PCR assays for *Streptococcus pneumoniae*.

Assay	*S. pneumoniae* genome copy number^a^							
	10^6^	10^5^	10^4^	10^3^	10^2^	10	1	0
Sp PCR^b^	+	+	+	−	−	−	−	−
Sp LAMP, 35 min^c^	+	+	+	+	+	+	−	−
Sp LAMP, 60 min^c^	+	+	+	+	+	+	−	−

a Results obtained by triplicate trial: +, amplification occurred; −, amplification did not occur.

b Results obtained by electrophoretic analysis.

c Results determined by visual inspection.

**Table 3:** Detection limit of the LAMP and PCR assays using cerebrospinal fluid specimens spiked with *S. pneumoniae* (ATCC 6305).

Assay	*S. pneumoniae* genome copy number^a^							
	10^6^	10^5^	10^4^	10^3^	10^2^	10	1	0
Sp PCR^b^	+	+	+	−	−	−	−	−
Sp LAMP, 60 min^c^	+	+	+	+	+	+	−	−

a Results obtained by duplicate trial: +, amplification occurred; −, amplification did not occur.

b Results obtained by electrophoretic analysis.

c Results determined by visual inspection.

### Preparation of Chromosomal DNA

Genomic DNA was purified from the 57 strains above using a QIAamp DNA mini kit (QIAGEN, Valencia, CA) according to the manufacturer’s protocol. For the detection limit analysis, genomic DNA from *S. pneumoniae* ATCC 6305 was obtained as previously described, and the concentration was determined using an Ultrospec 3300 Pro spectrophotometer (Amersham Pharmacia Biotech, Cambridge, UK). The number of genome copies was calculated based on the known genome molecular size (2.04 Mbp) of *S. pneumoniae* R6 (accession number AE007317). To ascertain the detection limit of the Sp LAMP assay, serial ten-fold dilutions of genomic DNA were amplified, and the results were compared with those obtained using conventional Sp PCR.

**Table 4:** Detection of *S. pneumoniae* by PCR and culture compared to detection by LAMP in 106 cerebrospinal fluid specimens.

Result	LAMP Result		Total (n = 106)
	Positive (n = 33)	Negative (n = 73)	
PCR-positive	18	0	18
PCR-negative	15	73	88
Culture-positive	11^a^	0	11
Culture-negative	22	73	95

a Every culture-positive specimen was also positive by Sp LAMP and Sp PCR.

For the detection limit study, triplicate Sp LAMP testing was performed over 3 days using the ten-fold dilutions of genomic DNA. Two technicians independently tested the same samples to confirm the reproducibility of Sp LAMP results. The supernatant of a pooled pneumococcus-negative CSF specimen [16] was used for a spiking assay in which serial ten-fold dilutions of genomic DNA were amplified, and the results were compared between Sp LAMP and conventional Sp PCR.

### Sp LAMP Primer Design

We designed a loop primer using LAMP primer support software (Net Laboratory, Kanagawa, Japan) based on published sequences of the *lytA* gene of a *S. pneumoniae* strain (GenBank accession number: AE008540) [14]. As described previously [14], we compared the *lytA* sequences of four *S. pneumoniae* strains (GenBank accession numbers: AE008540, AE007483, M13812 and AF467249) and nine other organisms that harbor the *lytA* gene, namely, *Streptococcus mitis lytA* (GenBank accession number: AJ617815 and AJ617816) and *Streptococcus* species *lytA* (GenBank accession numbers: AJ252190, AJ252191, AJ252192, AJ252193, AJ252194, AJ252195 and AJ252196). The consensus sequence among the *S. pneumoniae* strains was determined (Fig. 1), and *S. pneumoniae*-specific LAMP primers, specifically F3, B3, FIP, BIP, and LB (Fig. 1), were designed after the alignment analysis.

### Sp LAMP Reaction

The reaction mixture (25 µL) contained 1.6 µM each of FIP and BIP, 0.2 µM each of F3 and B3, 0.4 µM of LB, 8 U of Bst DNA polymerase large fragment (New England Biolabs, Ipswich, MA, USA), 1.4 mM deoxynucleoside triphosphates, 0.8 M betaine (Sigma, St. Louis, MO, USA), 20 mM Tris-HCl (pH 8.8), 10 mM KCl, 10 mM (NH_4_)_2_SO_4_, 8 mM MgSO_4_, 0.1% Tween 20, and template DNA (2 µL, for analytical determination purposes). The mixture was incubated at 63°C for either 35 or 60 minutes and then heated at 80°C for 2 minutes to terminate the reaction.

### Analysis of Sp LAMP Products

The LAMP reaction was evaluated by visual inspection because the Sp LAMP reaction generates turbidity proportional to the amount of amplified DNA [18]. Amplified products were resolved by electrophoresis in 3% agarose gels, followed by visualization by ethidium bromide staining. For further confirmation, a Loopamp real-time turbidimeter (LA-200; Teramecs, Kyoto, Japan) monitored the turbidity in the reaction tube in real time by reading the OD_650_ every 6 seconds. The presence of a greater quantity of initial template DNA shortened the threshold time to detection of *S. pneumoniae*. We used the application software for the turbidimeter to obtain the amplification time required to exceed the turbidity level of 0.1 (*Tt*), according to the manufacturers’ protocol [18].

To confirm the fidelity of amplification, a portion of the amplified products was digested with the restriction enzyme TasI (Fermentas Inc., Hanover, MD, USA), and their sizes were analyzed by electrophoresis in 3% agarose gels, followed by ethidium bromide staining. To verify the structure of the amplified Sp LAMP products, the amplified products were sequenced using a BigDye Terminator V3.1 cycle sequencing kit (Applied Biosystems, Foster City, CA, USA) and an ABI PRISM 377 DNA sequencer (Applied Biosystems) according to the manufacturer’s instructions. The primers used to sequence the target region (between F1 and B1) were as follows: F2 primer, 5′-CACTCAACTGGGAATCCGC-3′, and B2 primer, 5′-CCAGGCACCATTATCAACAGG-3′ (Fig. 1).

### PCR Assay

We used the sequences of conventional Sp PCR primers described previously for *lytA* [6], [7]. The PCR mixture (10 µl) consisted of 0.2 mM of each deoxyribonucleoside triphosphate, 10 mM Tris-HCl buffer (pH 8.3), 50 mM KCl, 2 mM MgCl_2_, 1 U of Ex Taq DNA polymerase (TaKaRa Bio Inc., Otsu, Japan), 0.5 µM concentrations of each primer and 1 µl of template DNA (sample volume was chosen for analytical determination purposes). PCR was performed using a thermal cycler (MJ Research, Waltham, MA, USA) for 30 cycles. Each cycle consisted of 15 s at 94°C, 15 s at 53°C, and 15 s at 72°C. Products were visualized by resolution on a 2% agarose gel followed by staining with ethidium bromide.

### Statistical Analysis

The clinical sensitivity and specificity of conventional Sp PCR and culture testing were compared to those of Sp LAMP (the gold standard was Sp LAMP results). A linear regression line was obtained by plotting the amplification time required to exceed the turbidity level of 0.1 (*Tt*) against the log of the initial template DNA.

## Results and Discussion

To develop a more rapid and sensitive Sp LAMP reaction, we substantially refined our previously reported Sp LAMP assay [14] by adding another loop primer (loop primer backward). With the addition of this primer, the Sp LAMP assay successfully amplified the 194-bp target sequence of the *lytA* gene locus within 30 min (previously 60 min [12]) (Fig. 2). The product was visible on an agarose gel (Fig. 3). The amplified product had a ladder-like pattern on the gel that is characteristic of the LAMP reaction and indicates the production of stem-loop DNAs with inverted repeats of the target sequence [11].

### Analytical Specificity of Sp LAMP

To evaluate the species specificity of the Sp LAMP primers, we tested 32 reference strains and 25 clinical alpha-hemolytic streptococcal strains, including four *S. pneumoniae* strains and 21 other *Streptococcus* species (three *S. oralis*, 17 *S. mitis*, and one *Streptococcus* species) known to carry the *ply* and *lytA* genes (Table 1). For each assay mixture, a standard genomic DNA concentration (10^6^ copies) was used for each strain. In the Sp LAMP reaction, amplification of pneumococcal DNA was observed after 35 minutes. In contrast, genomic DNA of non-pneumococcal strains was not amplified even after 60 minutes of incubation, whereas PCR produced four false-positive results for *lytA* and 17 false-positive results for *ply* in 25 clinical streptococcal strains (Table 1). Amplification specificity was confirmed by TasI digestion to ensure that the product contained sequences corresponding to the selected target gene sequence of the pneumococcus *lytA* gene locus. The products of TasI digestion were 107 and 108 bp, which is consistent with the predicted sizes (Fig. 3). Amplified products were further analyzed by sequencing, and the sequences were compared with those of the targeted region (bases 133 to 154 in the original sequence) of the pneumococcus *lytA* gene (between F1 and B1; Fig. 1). The sequences obtained were identical to the expected nucleotide sequences (data not shown). The Sp LAMP assay used in this study was more analytically specific than Sp PCR. Because the amplification reaction occurs only when all seven regions within a target DNA (Fig. 1) are correctly recognized by the primers [13], the Sp LAMP assay has high specificity. The higher analytical specificity of the Sp LAMP assay is in agreement with our previous findings [14], [19]. To improve the specificity of the LAMP reaction, it is important to locate more specific sequences within primer regions. Several primer sets were designed and tested for detection limit and analytical specificity by using reference DNA in optimal conditions, and the primer set described herein showed the best results.

### Detection Limit of the Sp LAMP Assay

Serial ten-fold dilutions of genomic pneumococcal DNA were reliably amplified at a lower limit of 10 genome copies per reaction (triplicate trial) (Table 2). In contrast, Sp PCR had a detection limit of 10^4^ (Table 2) genome copies per reaction. The detection limit of the Sp LAMP assay was identical when measured by the Loopamp real-time turbidimeter, direct visual inspection or gel electrophoresis (Table 2, Fig. 2A). Similarly, using spiked CSF specimens, the detection limit of the Sp LAMP assay was 10 genome copies and was 10^4^ genome copies for Sp PCR (Table 3). Overall, the detection limit of the Sp LAMP assay was 1,000 times lower than that of Sp PCR. No amplification was apparent in the Sp LAMP reaction when the sample tube lacked target DNA. Identical results were replicated in a triplicate experiment conducted over three days by two independent laboratory technicians.

Previous studies using the same Sp PCR assay and a real-time PCR assay that target the *lytA* gene were reported to have detection limits around 50 [20] and 22 copies [9] per reaction, respectively. The Sp LAMP assay (detection limit, 10 copies) was more sensitive than either conventional or real-time PCR. The lower detection limit of the Sp LAMP assay is in agreement with our previous findings [14], [19].

### Real-time Turbidity Measurement

As the LAMP reaction progresses, it generates pyrophosphate ions that bind to magnesium ions to form a white precipitate of magnesium pyrophosphate. The resulting turbidity is readily assessed by direct visual inspection. This characteristic feature of the LAMP reaction can be used to evaluate the reaction end-point simply by observing the presence of precipitate. Real-time turbidity measurements of the LAMP reaction [16] permit quantitative detection of minute amounts of nucleic acids with high precision and over a wide range. In the present study, using that apparatus, the curve of real-time turbidity measurements had high linearity from 10 to 10^6^ copies of *S. pneumoniae* template DNA with an excellent correlation coefficient (r^2^ = 0.999, Fig. 2B). This observation indicates that the concentration of any template DNA can be determined by comparing the *Tt* value with the *Tt* values of template DNA samples of known concentrations. Thus, when a real-time turbidity monitoring system is used, the concentrations of pneumococcal DNA in clinical samples can be quantified.

### Sp LAMP and Sp PCR Analysis of CSF Specimens

We tested 106 CSF specimens by Sp LAMP and Sp PCR assays. Among 33 Sp LAMP-positive specimens, 18 (54.5%) were Sp PCR positive and 15 were Sp PCR negative, and 11 CSF specimens were *S. pneumoniae* culture positive (33.3%) (Table 4). In addition, 73 Sp LAMP-negative specimens (100%) were also negative by Sp PCR and culture methods (Table 4). All 11 *S. pneumoniae* culture-positive specimens (100%) were positive by both Sp LAMP and Sp PCR assays.

Gram-positive cocci consistent with *S. pneumoniae* were identified by gram stain in 16 CSF specimens. Of these, 10 were positive by pneumococcal culture, Sp PCR and Sp LAMP; five were negative by pneumococcal culture, Sp PCR and Sp LAMP (including three *Streptococcus agalactiae* culture-positive and two coagulase-negative *Staphylococcus* culture-positive specimens); and one CSF specimen was negative by pneumococcal culture and Sp PCR but positive by Sp LAMP. The original culture of this CSF specimen showed positive growth, but the organism was not identified. Thus, it seems the lower detection limit of the Sp LAMP reaction affected this result.

We confirmed, by sequencing and digestion with TasI, that all of the Sp LAMP reaction products for CSF corresponded to the selected gene target (New England Biolabs, data not shown). Compared with Sp LAMP, the clinical sensitivity of Sp PCR and culture tests was 54.5% and 33.3%, respectively; and the clinical specificity of the two tests was 100%. Thus, the Sp LAMP assay was more sensitive than the Sp PCR and culture methods.

In our analysis, the observation that 23% (22/95) of the culture-negative CSF specimens and 17% (15/88) of the Sp PCR-negative CSF specimens were Sp LAMP-positive. From a clinical viewpoint, the excellent clinical sensitivity and specificity of the Sp LAMP assay will be valuable to confirm and exclude the presence of pneumococcal infection [21]. More accurate diagnosis of *S. pneumoniae* infection obtained using this Sp LAMP assay could improve the use of antibiotics and reduce currently high levels of antibiotic resistance. From a public health standpoint, widespread application use of the Sp LAMP assay may could yield higher detection rates and alter global pneumococcal prevalence rates in the future.

Our results showing higher clinical sensitivity of the Sp LAMP assay are consistent with results of the LAMP assay developed for detecting *H. influenzae* type b in CSF [19]. Previous studies demonstrated that the LAMP reaction is more tolerant of potentially perturbing biological substances than PCR [22]. In the present study, the detection limit for PCR assays, using spiked CSF specimens (Table 3), was the same as that obtained using purified *S. pneumoniae* DNA (Table 2). Thus, the PCR assays we used seemed to be well optimized for clinical CSF specimen testing and were minimally perturbed by biological substances. Overall, the main reason for higher clinical sensitivity of the LAMP assay seems to be higher analytical sensitivity.

In addition, the robust performance of the LAMP assay in previous studies [23] suggests that LAMP-based detection of *S. pneumoniae* and other invasive bacterial pathogens could be feasible in a wide variety of clinical settings. The cost (including cost of reagents, supplies and personnel time) of performing the Sp LAMP in our laboratory suggests that the per-specimen cost of the LAMP assay is remarkably lower than PCR. The low technology requirements for the LAMP assay suggest that this platform could be particularly suitable for resource-limited settings in many developing countries.

In conclusion, the Sp LAMP assay established in this study has a detection limit more sensitive than those previously described for Sp PCR methods, using both purified DNA and DNA-spiked CSF. The lower detection limit of the Sp LAMP assay yielded a higher detection rate for CSF specimens compared to the Sp PCR assay. Further evaluation of the Sp LAMP in prospective studies is now underway to confirm the Sp LAMP test’s characteristics, including clinical sensitivity, clinical specificity, predictive values and likelihood ratios compared with bacterial culture, antigen detection and PCR.
